# Scientometrics Analysis of World Scientific Research of Pathology and Forensic Medicine

**DOI:** 10.30699/ijp.2022.541660.2756

**Published:** 2022-02-20

**Authors:** Zeinab Jozi, Hamzehali Nourmohammadi

**Affiliations:** Scientometrics and Library & Information Sciences, Faculty of Humanities, Shahed University, Tehran, Iran

**Keywords:** Article citations, Bibliometrics, Citation analysis, Patent citations, Pathology and forensic medicine, Science-technology relationship

## Abstract

**Background & Objective:**

This study examines the extent of scientific publications and patents in pathology and forensic medicine globally and the citation relationship between them from 2011 to 2020, indexed in the Scopus database.

**Methods:**

In this scientometric study, data were extracted from the SciVal citation database. Search feature and library study method and annual growth rate, relative growth rate, and linear model were used to retrieve and analyze the data. The scientometric indicators included the number of publications and patents of the university in collaboration with industry, the number of articles cited by patents, the number of patents cited by articles, the average number of patents cited by articles, and the number of articles cited.

**Results:**

The results showed a poor collaboration between academia and industry in pathology and forensic Medicine, and North America is the busiest region in this field. The average growth of patents based on articles, the number of articles mentioned in patents, citations to patents, and the average number of patents of an institute in the articles of that institute have a positive exponential relationship. Based on the linear model, the relationship between articles and citations equals R^2^ = 0.982, which is inverse and negative. The data set of articles and citations was suitable for polynomial (R^2^ = .994), linear (R^2^= .982) and exponential (R^2^ = .887) models.

**Conclusion:**

The research process of pathology and forensic medicine is inappropriate, and the citation relationships between articles and patents in this field are weak. Strengthening the link between academia and industry in pathology and forensic medicine can strengthen research in this field.

## Introduction

Today, the trend towards the scientific and technological policy is increasing. The ability to generate scientific and technological knowledge (S&T) and translate it into new products or processes is a key tool for economic growth and development. It plays an increasingly important role in the economic develop-pment of countries worldwide (1). Scientific products and patents are the basis for the growth of science and technology, and indicators based on scientific products and patents are the main methods for assessing societies' progress, economic growth, and development and measuring the relationship between basic research and industry (2). A new approach to science and technology interactions can be achieved by analyzing patents and articles. Because analysis of patents citations provides information on previous patents citations and related articles, it is helpful to examine the process of techno-logy development, evaluate the relationship between scientific research and technology development, disseminate knowledge and understand the nature of inventions. Scientific research also plays an essential role in stimulating industrial innovations (3), and patents that receive more citations have high industrial value (4). One of the most common methods for citation analysis of scientific products and patents is scientometrics. Scientometrics is the science of measuring and analy-zing science using qualitative, quantitative, and com-putational approaches. Scientometrics, with its various indices, is a reliable method for evaluating scientific development (5). In addition to evaluating and investi-gating all aspects of scientific literature, scientometrics examines hidden relations and connections within scientific fields and subfields through citation analysis methods (6).

Research in medicine has received much attention due to its economic profitability among the necessary research fields, as medical research is related to human longevity and health. One of the disciplines of medicine as an important bridge between the basic and clinical sciences of biomedicine is the field of pathology and forensic Medicine, which is helpful in diagnosis, treatment, and prevention (7). Pathologists provide the link between the clinical aspect and the natural sciences of medicine. They neither see the patient nor treat them. After diagnosing the disease, they refer the patient to close counselors with medical contact. These extensive and close interactions with various medical disciplines demonstrate the importance of pathologists' work in the clinical-therapeutic and diagnostic relationship and demonstrate the involvement of pathologists in research projects by clinical colleagues (8).

Previous studies such as study by Brusoni, Criscu-olo, and Geuna, measuring the citations between patents and non-patents from 30 major pharmaceutical groups, found that the study groups gradually increased the breadth of their knowledge base and moved toward appropriate areas for new biopharmaceutical research (9). Szu-chia examining the number of citations to patent articles in the field of genetics found that the development of technology in genetic engineering is strongly influenced by research conducted by the public sector, with more than 90% citations by non-patent sources and others. More than 67% of patent journal articles are owned by US companies, and a further survey of corporate authors shows that patents belong to academic authors (10). Nourmohammadi examination of the situation in Iran, Turkey, Saudi Arabia, India, Pakistan, South Korea, and South Africa and the presence of these countries in the field of pathology and forensic medicine showed that these countries are different based on "citing any evidence". Because the "number of documents" and "total number of citations" of their articles do not match, and therefore the citation to each document does not increase (11). Bousfield *et al.,* measuring citations in more than 8,000 biomolecular patents, showed that citations in the biomedical literature and patents have a different pattern (12). Xu *et al.,* after examining 329 US patents and citing them in the field of AD, concluded that the patent citation network is very fragmented (13). Emami, Riahinia, and Soheili, to exa-mine the relationship between science and technology in the field of medical and laboratory equipment, showed that the number of inventions and scientific articles published in the field of medical and laboratory equipment has steadily increased during the review period. Also, the number of patents and scientific articles in this field of research has grown significantly in a given period. The rate for citing US patents for medical and laboratory equipment patents is much higher than for citing non-US patents and other types of patents (14). In examining how to release the master's thesis in the field of pathology, KALA *et al.* determined that only one article among all published articles has a citation rate of 0.5 per year and the average citation rate is 0.02 per article. According to the results of this study in the field of anatomical pathology, the very low citation rate had questioned the quality of research in this field (15). In a study by Ingrole and Azizoglu, on microneedles, the number of articles (more than 1,000), 750 patents and nearly 80 clinical trials showed strong and growing microneedle activity. This technology is rapidly evol-ving and is being used for new applications for the benefit of human health and well-being (16). 

Recent studies in medical research show the need to improve collaboration among private and public sectors and health care organizations in research to achieve scientific innovation through joint research (17). This type of analysis of scientific texts and patents has shown that, for example, increasing knowledge in biomedical research has led to the growth of research in this field, but the development of technology in genetic engi-neering is still dependent on the public sector, and most authors in this field. Therefore, some disciplines have a strong relationship between science and technology, and others have an unstable relationship in this field. Given that research has not yet evaluated the field of pathology and forensic medicine and the relationship between scientific literature and research in this field, the purpose of this study is to evaluate the process of pathology and forensic medicine research, including the collaboration rate of university and industry, and to investigate the relationship between science and technology through the number of articles cited by patents, the number of patents cited by articles, the number of citations to patents, the average of patents citing articles of the same institute and the amount of scientific productions and citations to scientific productions that has been indexed in the Scopus database. Reviewing and evaluating such research not only helps medical professionals grow their careers but also gives them a good reputation in the field as well as the institution in which they operate. Even from the evaluation of such research, it can be understood why and how an individual or organization may qualify for any kind of grant, honor, or award (18).

## Material and Methods

**Scientific Findings**

In this descriptive-quantitative study, the world scientific research in the field of pathology and forensic medicine from 2011 to 2020 was reviewed in the Scopus citation database. The data collection method was the documentary study conducted on August 29, 2021, using the SciVal database. After collecting data on the number of university-industry collaborations, the number of articles cited in patents, the number of patents cited in articles, the number of citations to patents, the average number of patents mentioned in articles by each institution, and the number of citations of articles were entered in Excel software and then analyzed using scientometric techniques to calculate the annual growth rate, relative growth rate, and growth models.

**Analysis**

**Scientific Indicators**

For annual growth, we presented data as the number of retrieved documents each year. Furthermore, the annual growth rate (AGR), defined as the percentage change in the number of publications over a period of 1 year, was calculated based on the following equation: AGR= [(Ending Value - Beginning Value)/Beginning Value] * 100 (19).

$\mathrm{AGR}=\frac{\mathrm{Ni}-\mathrm{Nj}}{\mathrm{Nj}}\mathrm{*100}$

Growth of publications, patents, citations, and growth rate indicators were analyzed based on two scientometric parameters, namely, the relative growth rate (RGR) and doubling time (DT). RGR is the increase in the number of publications, citations, and patents per unit of time, and it is calculated using the formula RGR (20): 

 (1)

$\mathrm{Relative Growth Rate}\left(\mathrm{RGR}\right)=\frac{\mathrm{LN}(N2)-\mathrm{LN}(\mathrm{N1})}{\mathrm{T2}-\mathrm{T1}}$

Where,

RGR = N1 and N2 = Loge (are the cumulative numbers of publications for years T2 and T1)

T1 = The unit of the initial time 

T2 = The unit of the final time

Since the current study calculates the RGR for successive years and given that T2, T1 = 1, Eq. (1) can be simplified as Eq. (2):

(2)

 RGR= LN (N_2_) – LN (N_1)._

Dt, on the other hand, is calculated as Eq. (3)

(3)

$\mathrm{Doubling time}\left(\mathrm{Dt}\right)=\frac{\left(\left(\mathrm{T2}-\mathrm{T1}\right)\mathrm{*LN2})\right)}{\mathrm{RGR}}$

**Growth Model**

Growth models were used to identify the linear and exponential trend of citations to articles. According to this method, the closer R^2^ is to the number 1, the trend follows the linear trend, and the closer it is to the number 0, it gets away from the linear trend (21)**.**

## Results

**Fig. 1:**
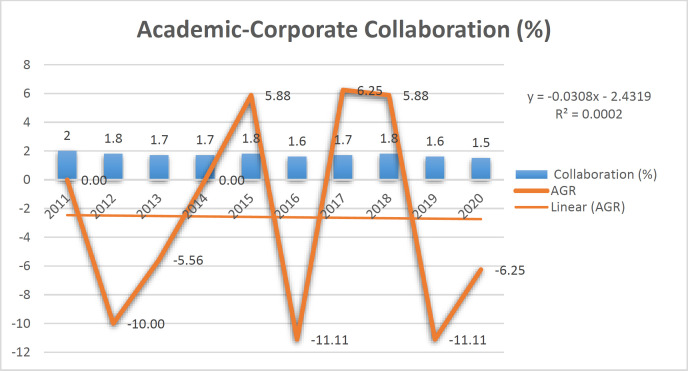
Academic-Corporate Collaboration (%)

It was found that the collaboration of universities with industry in pathology and forensic medicine in the world is decreasing. According to Figure 1, the university's collaboration with industry is low in the number of scientific publications and patents, so the highest number has been registered for 2011. After shallow ups and downs from 2012 to 2019, it showed fluctuations in publications. In 2020, 1.5% collab-oration was the lowest. However, a study on the growth rate of collaboration between the two institutions (University and industry) revealed that the highest annual growth rate of collaboration was in 2015, 2017, and 2018 with 6.25, and the lowest collaboration in 2016 and 2019 is -11.11. Based on the linear relationship, it was found that the linear trend of scientific production growth equals R^2^ = 0.0002. 

**Fig. 2:**
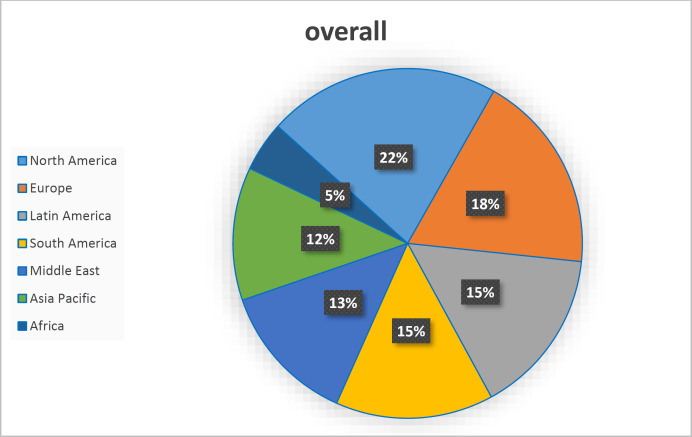
Academic-Corporate Collaboration (%)

This study examined the level of collaboration of the world's universities in different regions. According to Figure 2, North America has 22% collaboration. The European continent has 18%, and South America and Latin America have 15% collaboration. The Middle East has 13%, Asia and Oceania for 12%, and Africa has only 5% collaboration with industry in pathology and forensic medicine.

Table 1 shows the number of patents citing articles from institutions retrieved from the Scopus database in the field of pathology and forensic medicine during the period 2011 to 2020. The growth rate has been declining, from 8.19 in 2011 to 2.46 in 2020. The maximum relative growth of was recorded at 6.89 in 2020. The average relative growth rate was 2.74, and the doubling time was 0.30 from 2011 to 2020. Therefore, the trend of relative growth rate is increasing, and the trend of doubling time is linear. 

**Table 1:** Number of Citing-Patents Count of Institutions in the Field Pathology and Forensic Medicine

year	NO. of Citing-Patents Count	Cumulative NO. of Citing-Patents Count	loge(1)p	loge(2)p	RGR(p)	Mean RGR (p)	Dt(p)	Mean Dt(p)
2011	3603	3603	8.19	8.19	0	2.76	0	**0.30**
2012	2956	6559	7.99	8.79	0.80		0.87	
2013	2258	8817	7.72	9.08	1.36		0.51	
2014	1669	10486	7.42	9.26	1.84		0.38	
2015	1428	11914	7.26	9.39	2.12		0.33	
2016	893	12807	6.79	9.46	2.66		0.26	
2017	579	13386	6.36	9.50	3.14		0.22	
2018	282	13668	5.64	9.52	3.88		0.18	
2019	100	13768	4.61	9.53	4.92		0.14	
2020	14	13782	2.64	9.53	6.89		0.10	
	**13782**					

According to our findings, the growth rate of the number of publications cited by patents has been decreasing. It reached 6.66 in 2011 to 2.56 in 2020 (Table 2). Also, the maximum relative growth rate is 5.64, and its minimum is 0.75. The trend of the growth rate of doubling time is linear. The average relative growth rate is the number of publications cited by patents in the field of pathology and forensic medicine globally is equal to 2.41, and the average rate of doubling time is 0.33.

**Table 2:** Number of Patent-Cited Scholarly Output by World Patents Pathology and Forensic Medicine

Year	No. of Patent-Cited Scholarly Output	Cumulative. NO. of Patent-Cited Scholarly Output	Loge(1)p	Loge(2)p	RGR(p)	Mean RGR (p)	Dt(p)	Mean Dt(p)
2011	784	784	6.66	6.66	0.00	2.41	0.00	**0.33**
2012	703	1487	6.56	7.30	0.75		0.93	
2013	590	2077	6.38	7.64	1.26		0.55	
2014	461	2538	6.13	7.84	1.71		0.41	
2015	435	2973	6.08	8.00	1.92		0.36	
2016	279	3252	5.63	8.09	2.46		0.28	
2017	221	3473	5.40	8.15	2.75		0.25	
2018	129	3602	4.86	8.19	3.33		0.21	
2019	50	3652	3.91	8.20	4.29		0.16	
2020	13	3665	2.56	8.21	5.64		0.12	
	**3665**					

The number of citations to institutions' patents is expressed in Table 3. Based on this table, the cumulative frequency of citations and growth rate, relative growth rate, and doubling time were examined. The growth rate of Patent citations showed that the amount of citations is gradually decreasing. The related growth rate in this index is also increasing so that it had increased from 0.81 in 2012 to 6.96 in 2020. Basic estimates have a significant impact regarding citation to patents and the level of its effectiveness. According to the findings, the average relative growth rate of 15373 citations equals 2.74, and the average doubling time is 0.30.

**Table 3:** Number of Patent-Citations Count of Institutions in the Field Pathology and Forensic Medicine

year	No. of Patent-Citations Count	Cumulative. NO. of Patent-Citations Count	loge(1)p	loge(2)p	RGR(p)	Mean RGR (p)	Dt(p)	Mean Dt(p)
2011	4084	4084	8.31	8.31	0.00	2.74	0.00	**0.30**
2012	3259	7343	8.09	8.90	0.81		0.85	
2013	2610	9953	7.87	9.21	1.34		0.52	
2014	1843	11796	7.52	9.38	1.86		0.37	
2015	1538	13334	7.34	9.50	2.16		0.32	
2016	1208	14542	7.10	9.58	2.49		0.28	
2017	690	15232	6.54	9.63	3.09		0.22	
2018	376	15608	5.93	9.66	3.73		0.19	
2019	113	15721	4.73	9.66	4.94		0.14	
2020	15	15736	2.71	9.66	6.96		0.10	
	**15736**					

Citation analysis of the number of patents of an institution that cite to the scientific products of that institution in Table 4 showed that the inventors of these universities in early 2011 cited only 5.59% to their university articles. As the growth rate shows, this amount has been decreasing, and for 2020, the number -0.36 has been recorded. Also, the related growth rate in this index showed that the average of patents citation to university articles has increased. Here, the average related growth rate is 2.81, and the doubling time in this index is 0.29 per year. According to these results, the inventors' citation tendency to the origin university articles takes 0.29 time to reach a doubling growth rate. 

The annual Fit Curve of citation to articles from 2011 to 2021 based on Figure 4 confirms its exponential nature (22). (R^2^=0.982) This means that the number of citations has decreased with the increase of articles and shows a negative and inverse relationship. 

**Table 4:** The Patent-Citations per Scholarly Output by the Institute to that Institute Article in the Field of Pathology and Forensic Medicine

year	No. of Patent-Citations per Scholarly Output	Cumulative. NO. of Patent-Citations per Scholarly Output	loge(1)p	loge(2)p	RGR(p)	Mean RGR (p)	Dt(p)	Mean Dt(p)
2011	268.9	268.9	5.59	5.59	0.00	2.81	0.00	**0.29**
2012	203.7	472.6	5.32	6.16	0.84		0.82	
2013	161.4	634	5.08	6.45	1.37		0.51	
2014	108.1	742.1	4.68	6.61	1.93		0.36	
2015	84.1	826.2	4.43	6.72	2.28		0.30	
2016	68.3	894.5	4.22	6.80	2.57		0.27	
2017	39.3	933.8	3.67	6.84	3.17		0.22	
2018	22.4	956.2	3.11	6.86	3.75		0.18	
2019	6.5	962.7	1.87	6.87	5.00		0.14	
2020	0.7	963.4	-0.36	6.87	7.23		0.10	
	**96.34**					

**Fig 3:**
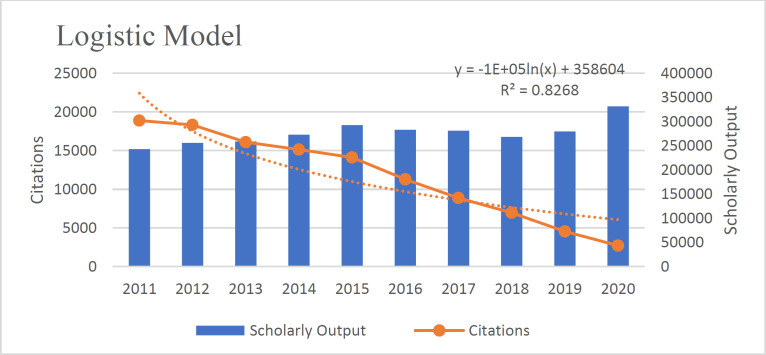
The Logistic model fit of the relationship between articles and citations received in the field of pathology and forensic medicine

**Fig. 4:**
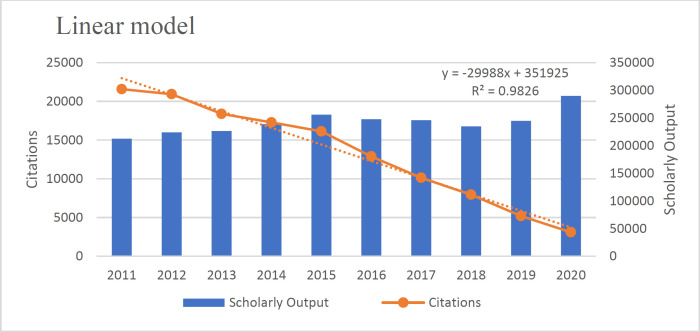
The Linear model fit of the relationship between articles and citations received in the field of pathology and Forensic medicine

**Fig. 5:**
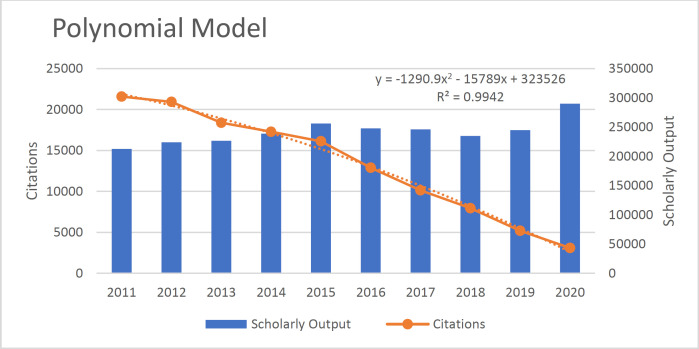
The polynomial model fit of the relationship between articles and citations received in the field of pathology and forensic medicine

**Fig. 6:**
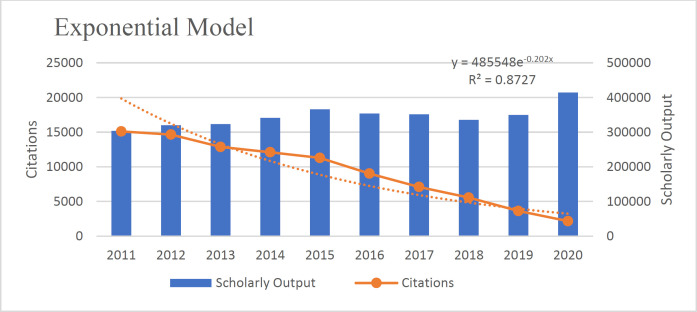
The exponential model fit of the relationship between articles and citations received in the field of pathology and forensic medicine

**Fig. 7:**
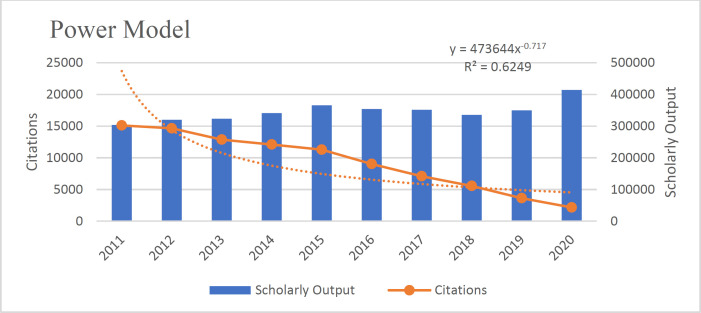
The power model fit of the relationship between articles and citations received in the field of pathology and forensic medicine

According to the results, the Polynomial Model is more suitable than the linear model (R^2^=0.982). The existing data fits the Polynomial Model (R^2^=.994), Linear Model (R^2^=0.982), Exponential Model (R^2^=.887), Logistic Model (R^2^=0.826), and Power Model (R^2^=0.659) well because the value of their R^2 ^was close to 1(Figure 3-7).

## Discussion

This study analyzed the process of university-industry collaboration and citations to scientific products and patents in pathology and forensic medicine based on Scopus data from 2011 to 2020. According to the extensive studies that have analyzed the collaboration between university-industry, the relationship between university-industry has been beneficial in developing clinical research and medical devices and equipment (23). It provides access to technical and professional support and increases the necessary facilities for research and development activities. In addition, collaborations enable resear-chers to understand better the nature of companies' scientific needs (24). The data of this study showed the opposite of these statements. The growth rate of the collaboration of universities with industry in the field of pathology and forensic medicine fluctuates, and after a positive growth, it has taken a negative change. The linear growth trend showed that the level of collaboration between the university and industry does not follow the linear trend. This may be due to the lack of proper infrastructure in collaboration between the university and industry or the lack of sufficient funds to equip laboratories in pathology and forensic medicine. North America is also the most active region globally, and the African continent has the lowest activity. This showed the interest of North American researchers in pathology and forensic medicine. Although clinical research has evolved significantly in the last two decades, it seems that research conducted by academics is in conflict with industry, and the primary goals of R&D are clearly different between industry and academia. That is why many researchers in this field do not reach their goals (25). Industry and academia must be increasingly involved in pathology and forensic medicine to improve new devices and new treatment methods for the benefit of patients and health care by designing and advancing new ones and using both parties' knowledge, skills, and expertise to achieve a diagnosis.

The study's findings confirm the research results of Emami, Riahinia, and Soheili (14), who pointed to the positive relationship between citation to scientific texts and the technical efficiency of patents. In this study, through the relative growth rate, it was found that the number of patents citing articles is increasing, and the time for doubling patents is decreasing. An average of 2.74 for related growth rate and 0.30 for doubling time was obtained. This means that it takes 0.30 time for the number of patents citing articles to be doubled. Nevertheless, the fact is that citations to articles by patents in the field of pathology and forensic medicine continue to be neglected. 

This study also has examined citation performance regarding the number of articles cited by patents. In this regard, the results of this research and Brusoni, Criscuolo, and Geuna's (9), research indicated the prominence of articles cited by patents in terms of other bibliometric indicators. Bousfield *et al.* (12) showed that citation is a good indicator for examining the link between science and technology. According to their statements and the findings of this study, articles cited in patents can receive more citations from other articles. Because of the relative growth trend of related growth rate increases the number of articles cited by patents. The average related growth rate is 2.41, and the average doubling time of this growth is 0.33. However, during the years under review, the articles in pathology and forensic medicine have been neglected by patents. 

Citing patents have quickly become a standard in innovation quality (26). Because citation in patents can be used as an essential factor in assessing the relationship between science and industry (27), this article has considered the citations to patents as an essential factor in improving the performance of quality-producing processes in innovation. The related growth rate test results showed that the citation to patents had grown slightly. Furthermore, this index's average related growth rate is 74.2, and the time req-uired to reach double growth is 0.30. These results suggest that examining the patent citations may help conduct future research and organizations researching this area.

We also analyzed the evidence contained in citations by patents of an institution that cites the articles of the same institution. Our findings in this study match Wang and Guan's research (28); they stated the patents that are produced by universities and are moved toward scientific knowledge often cite to more scientific sources. However, according to de Almeida *et al.*, the citation is an important factor in the flow of knowledge in the organization, and organi-zations with high self-citation have a high capacity to attract capital and achieve high returns in attracting their previous investments (29). But according to the findings of this citation study, the citations conducted by patent inventors (patents owners) to the same institute's articles have slightly increased each year based on related growth rates. However, the average of this growth is 2.81, and it takes 0.29 time to double. The fact is that according to growth rate, citing the articles of an institution by the patents of that institution has a significant decrease; it reached -0.36 in 2020. Perhaps the reason for this is the reduction of inven-tions of institutions in the field of pathology and foren-sic medicine. Alternatively, it is why the subjects of the patents were unrelated to that institution's articles, or the articles were not qualitatively suitable for receiving a citation. These important factors reduce the value of scientific products in the field of pathology and forensic medicine in organizations and challenge the organization's efficiency.

This study also used the linear model approach to compare and cite scientific products. Analysis of scientific articles' citations is an essential tool in quantitative studies of science and technology (30). The number of citations that an article receives is considered representative of its scientific impact (31). Our study also emphasizes the research findings of Nourmohammadi, which showed that the countries under study were in a different situation in terms of "citation to each document" (11). As the number of documents and citations did not match, the citation to each document did not increase. The linear models showed how articles' growth and the citation to them were clear. There was a negative correlation between the two indices; although publications had increased, citations had reversed the linear trend. The study of linear models also showed that since all linear models were close to 1, they were all approved. 

## Conclusion

Considering the issue of human health and all research in medical sciences, especially pathology and forensic medicine, according to the present study results, pathology and forensic medicine research has not been strengthened as needed in the world. Therefore, it is necessary to strengthen the collabo-ration with industry, allocate more funds to research, increase the quality of products and make more efforts to apply scientific and technological products in this field.

According to the results obtained in this study, it is suggested:

- The number of scientific products and patents in pathology and forensic medicine resulting from the cooperation of universities and industry and citation relationships between them be examined;
- The top authors, inventors, organizations, and countries in the field of pathology and forensic medicine be examined, and their scientific collaboration mapping be drawn.

Also, in future studies, using data mining techno-logy, the importance of the studied subjects in scienti-fic products and patents in the field of pathology and forensic medicine be identified.

## Conflict of Interest

The author(s) declared that they have no potential conflicts of interest with respect to the research, authorship, and/or publication of this article.

## Funding

This study was supported by the National Institute for Medical Research Development (NIMAD), Islamic Republic of Iran.
